# Prognostic significance of tumor infiltrating immune cells in oral squamous cell carcinoma

**DOI:** 10.1186/s12885-017-3317-2

**Published:** 2017-05-26

**Authors:** Juan Fang, Xiaoxu Li, Da Ma, Xiangqi Liu, Yichen Chen, Yun Wang, Vivian Wai Yan Lui, Juan Xia, Bin Cheng, Zhi Wang

**Affiliations:** 1 Guangdong Provincial Key Laboratory of Stomatology, Guanghua School of Stomatology, Sun Yat-Sen University, No. 56, Lingyuanwest Road, Guangzhou, Guangdong 510055 China; 2 School of Biomedical Sciences, Faculty of Medicine, The Chinese University of Hong Kong, Hong Kong, SAR China

**Keywords:** Tumor infiltrating immune cell, Oral squamous cell carcinoma (OSCC), Prognosis, Overall survival (OS)

## Abstract

**Background:**

Prognostic factors aid in the stratification and treatment of cancer. This study evaluated prognostic importance of tumor infiltrating immune cell in patients with oral squamous cell carcinoma.

**Methods:**

Profiles of infiltrating immune cells and clinicopathological data were available for 78 OSCC patients with a median follow-up of 48 months. The infiltrating intensity of CD8, CD4, T-bet, CD68 and CD57 positive cells were assessed by immunohistochemistry. Chi-square test was used to compare immune markers expression and clinicopathological parameters. Univariate and multivariate COX proportional hazard models were used to assess the prognostic discriminator power of immune cells. The predictive potential of immune cells for survival of OSCC patients was determined using ROC and AUC.

**Results:**

The mean value of CD8, CD4, T-bet, CD68 and CD57 expression were 28.99, 62.06, 8.97, 21.25 and 15.75 cells per high-power field respectively. The patient cohort was separated into low and high expression groups by the mean value. Higher CD8 expression was associated with no regional lymph node metastasis (*p* = 0.033). Patients with more abundant stroma CD57^+^ cells showed no metastasis into regional lymph node (*p* = 0.005), and early clinical stage (*p* = 0.016). The univariate COX regression analyses showed that no lymph node involvement (*p* < 0.001), early clinical stage (TNM staging I/II vs III/IV, *p* = 0.007), higher CD8 and CD57 expression (*p* < 0.001) were all positively correlated with longer overall survival. Multivariate COX regression analysis showed that no lymph node involvement (*p* = 0.008), higher CD8 (*p* = 0.03) and CD57 (*p* < 0.001) expression could be independent prognostic indicators of better survival. None of CD4, T-bet or CD68 was associated with survival in ether univariate or multivariate analysis. ROC and AUC showed that the predictive accuracy of CD8 and CD57 were all superior compared with TNM staging. CD57 (AUC = 0.868; 95% CI, 0.785–0.950) and CD8 (AUC = 0.784; 95% CI, 0.680–0.889) both provided high predictive accuracy, of which, CD57 was the best predictor.

**Conclusion:**

Tumor stroma CD57 and CD8 expression was associated with lymphnode status and independently predicts survival of OSCC patients. Our results suggest an active immune microenvironment in OSCC that may be targetable by immune drugs.

## Background

Oral squamous cell carcinoma (OSCC) is a major cause of morbidity and mortality in patients with head and neck cancer. Even with multi-modality treatment, only modest improvement of patient survival has been reported. To date, the prognosis of OSCC patients remains unsatisfactory, as indicated by the poor 5-year survival rate of less than 20% in advanced patients [1–3]. Such a poor survival in OSCC patients indicates not only the aggressiveness of this cancer, but also insufficient understanding of the disease, which hinders the development of effective treatments. Our recent understanding of the involvement of immune components in disease progression, prognostic or treatment stratification in other cancers revealed the significance of immuno-characterization of human malignancies [4, 5], which is lacking in OSCC. Our current TNM staging system for OSCC is informative for prognosis, however, it is likely that additional immuno-characterization of OSCC tumors in situ may further facilitate treatment stratification, especially for the new arrays of immune drugs for cancer [1].

Tumor infiltrating immune cells have been shown to provide prognostic values in several human malignancies [6–10]. For adaptive immune cells, CD8^+^ cytotoxic lymphocytes (CTLs) were generally considered as the main force against cancer. Both intra- and peri-tumoral CD8 expression have been shown to predict better survival in colorectal and esophageal cancers [11–13]. CD4^+^ cells consist of several subpopulations and its benefit was controversial [14–17]. Among these subpopulations, Th1 is considered as a critical component of tumor surveillance. T-bet is commonly used as a specific marker of Th1 cells, its expression have been shown significantly associated with survival of patients with breast or gastric cancer [18, 19]. As the important components of innate immunity, functions of macrophages and natural killer (NK) cells in tumor microenvironment draw much attention in recent decades [20–22]. CD68 has been widely used as a pan-macrophage marker, its expression was reported associated with poor prognosis in breast cancer and hepatocellular cancer [23, 24]. CD57 expression is most prominent in highly mature cytotoxic NK cells and terminally differentiated effector T-cells such as CTLs and Th cells [25]. NK cells were important effectors of both innate and adaptive immune response, their killing capacity against tumor cells enhances in absence of MHC class I molecules. Tumour CD57 expression has been reported independently predicting survival in patients with colorectal cancer, gastric cancer and prostate cancer [26–28].

Yet, the precise role of immune cells in OSCC remained poorly defined and controversial, though primary OSCC tumors are known to be heavily infiltrated with lymphocytes [29]. Thus, in this study several representative immune subsets (CD8, CD4, T-bet, CD68 and CD57) in a cohort of surgically treated OSCC patients were determined. To keep the consistency and reproducibility, the methodology recommended by an international breast cancer TILs Working Group was tested [30]. This study will provide new strategies to select the most promising immune markers for clinic trials through an integrative scope.

## Methods

### Study population

Our study randomly enrolled 78 OSCC patients who underwent curative operations at the Department of Oral-Maxillofacial Surgery, Stomatology Hospital Affiliated to Sun Yat-sen University, China, between 2007 and 2009. TNM staging was determined according to the Union for International Cancer Control 2002 standard (UICC2002). Pathological examination was performed by two independent pathologists according to the 2005 revised World Health Organization classification of OSCC tumors. Ethical approval was obtained from the Ethical Review Committee of Guanghua School of Stomatology, Sun Yat-sen University, China. Written informed consents had been obtained from all patients. The data were analyzed anonymously.

### Immunohistochemistry

Paraffin-embedded specimens were cut into 4 μm thick sections. The slides were dewaxed by heating at 60 °C for 60 min followed by deparaffinizationin xylene and rehydration in graded alcohol. The slides were then put in Citrate Buffer solution (pH 6.0) and microwaved for 10 min at low power for antigen retrieval. Deparaffinized sections were stained with the following antibodies: CD8 1:150 (Abcam ab17147), CD4 1:250 (Abcam ab846), T-bet 1:100 (Abcam ab91109), CD68 1:100 (Dako M0814), CD57 1:150 (Abcam ab82749), Isotype Control (Abcam ab91353). The slides were then incubated in 3% H_2_O_2_ for 20 min for removal of endogenous peroxidase activity and subsequently incubated with secondary antibody (DAB) at 37 °C for 30 min. The tissue sections were immersed in a solution of 3, 3′-diaminobenzidine tetrahydrochloride (Dako, Hamburg, Germany) and then counterstained with hematoxylin.

### Microscopic evaluation of tumor sections

By use of a standard light microscope, images were acquired with a CCD-camera using a 20× objective, transferred to a PC and semi-automatically evaluated using the image analysis software COUNT (Biomas, Erlangen, Germany). Cases were scored blindly with respect to patient history, presentation, and previous scoring by two independent observers. In case of discrepancy, a final decision was made upon further re-examination of the slides in a microscope based on consensus by both pathologists.

Immune cells were identified by their specific markers (CD8, CD4, T-bet, CD68 and CD57). For each section, 10 areas of a representative field of tumor were assessed using an ocular grid comprising a high-power field (HPF) area of 0.0314 mm^2^. Tumor areas were divided into three anatomic compartments (i.e. tumor epithelial, tumor stroma and advancing tumor margin). The total number of each type of immune cells in tumor stroma, excluding cells within tumour cell nests, was counted. The average number of 10 HPFs was calculated as the final density of each section (cells per hpf).

### Follow up

Phone interviews and physical examinations of each patient were carried out once every 6 months. Patients were followed till the closing date of the study or death, whichever reached first. Overall survival (OS) was determined based on the date of diagnosis until the date of death or the end of study. All patients were followed for more than two years, the median follow-up time was 48 months (ranged from 29 to 93 months).

### Statistical analysis

All statistical analyses were performed using SPSS 16.0 software (SPSS, Chicago, IL, USA) and Stata/MP 14.0 software (Stata Corp, College Station, TX). Mean number of each type of immune cells was used as the cut off value separating patients into low and high infiltrated groups. Chi-square test was used to compare immune markers expression and clinicopathologic parameters. Overall survival (OS) was evaluated using the Kaplan–Meier method and the differences between survival curves were tested for statistical significance using the log-rank test. The Cox proportional hazards model was used to estimate the independent prognostic factors for OS. Receiver Operating Characteristic (ROC) and area under curve (AUC) were used to evaluate and compare the prognostic value of immune cells. All tests were two-sided and *p* < 0.05 was considered statistically significant.

## Results

### Patients outcome

The retrospectively registered cohort of 78 patients with OSCC who underwent a primary resection of tumor was investigated. Basic information and clinicopathological variables are summarized in Table 1. The average age of the patients was 60 years (range 24–82). The median follow up duration was 48 months (Inter-quartile range, 29–93 months). The panel consisted of about equal numbers of Stage I/II (*n* = 36, 46.15%) and Stage III/IV (*n* = 42, 53.85%) tumors. 48 (61.54%) patients were with lymphnode metastasis.

**Table 1:** Clinicopathological characteristics of OSCC patients

Factors		Number (range)	Percentage (%)
Age	Mean (range)	60 (24–82)	
Gender	Male	57	73.08
	Female	21	26.92
Smoking	Yes	47	60.26
	No	31	39.74
Drinking	Yes	42	53.85
	No	36	46.15
Differentiation	Well	57	73.08
	Moderate or poor	21	26.92
T stage	T1 and T2	58	74.36
	T3 and T4	20	25.64
N stage	N0	48	61.54
	N1-N3	30	38.46
Clinical stage	I and II	36	46.15
	III and IV	42	53.85

### The distribution of CD8^+^, CD4^+^, T-bet^+^, CD68^+^ and CD57^+^ cells in OSCC tissues

**Fig. 1:**
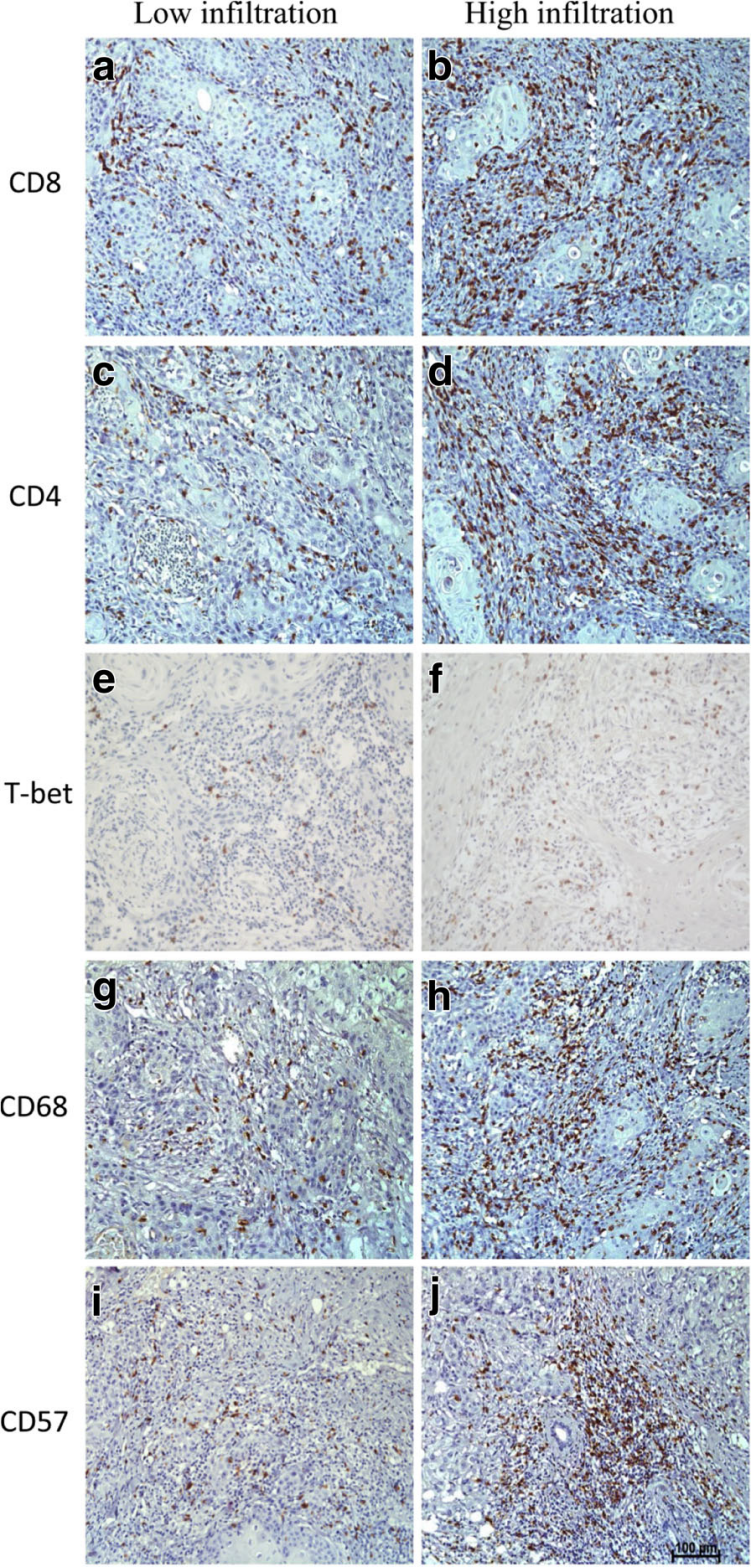
IHC analysis of immune cells distribution in OSCC tissues (200×). Immune cells were primarily distributed in tumor stroma. **a**–**b**: Representative images of IHC for evaluating low and high CD8 expression. **c**–**d**: CD4 expression; **e**–**f**: T-bet expression; **g**–**h**: CD68 expression; **i**–**j**: CD57 expression. **a**, **c**, **e**, **g**, **i**: Low expression; **b**, **d**, **f**, **h**, **j**: High expression

Positively stained immune cells demonstrated brown granules on the membrane. The majority of immune cells were located in stroma compartment around cancer nests. Only a few were detected in the center of nests. The mean number of each cell type was shown in Table 2. CD8, CD4, T-bet, CD68 and CD57 expression in low and high infiltrated groups was shown in Fig. 1.

**Table 2:** Mean numbers of immune cell in tumor stroma for 78 OSCC samples

	CD8	CD4	T-bet	CD68	CD57
Mean (SD) cells/hpf	28.99(12.67)	62.06(21.33)	8.97(3.99)	21.25(6.01)	15.75(9.41)
Range	8–65	9–104	0–18	7–32	3–62

### Relationships between clinicopathological features and density of immune cells

The relationships between clinicopathological features and density of immune cells were shown in Table 3. In the whole series, CD57 expression was associated with features of better prognosis including no lymphnode metastasis (*p* = 0.005), and early clinical stages (I/II vs III/IV, *p* = 0.016). Higher CD8 expression was significantly correlated with no lymphnode status (*p* = 0.033) and no drinking history (*p* = 0.014). T-bet was more abundant for patients older than 60 years (*p* = 0.046). CD4 and CD68 expression were not associated with any of the clinicopathological features in our OSCC cohort.

**Table 3:** Relationships between clinicopathological parameters of OSCC and densities of tumor infiltrating immune cells

Factors	CD8			CD4			T-bet			CD68			CD57		
	Low	High	*P*-Value	Low	High	*P*-Value	Low	High	*P*-Value	Low	High	*P*-Value	Low	High	*P*-Value
Age															
< 60	18	17	0.981	17	18	0.821	21	14	0.046*	21	14	0.238	23	12	0.137
≥ 60	22	21		22	21		16	27		20	23		21	22	
Gender															
Male	31	27	0.517	29	29	1	28	30	0.802	30	28	0.802	32	26	0.709
Female	9	11		10	10		9	11		11	9		12	8	
Smoking															
Yes	27	21	0.27	25	23	0.644	23	25	0.915	24	24	0.569	28	20	0.667
No	13	17		14	16		14	16		17	13		16	14	
Drinking															
Yes	27	15	0.014*	23	19	0.367	21	21	0.626	24	18	0.385	26	16	0.294
No	13	23		16	20		16	20		17	19		18	18	
Differentiation															
Well	26	31	0.101	31	26	0.205	27	30	0.984	28	29	0.319	32	25	0.937
Moderate or Poor	14	7		8	13		10	11		13	8		12	9	
T stage															
T1 and T2	29	29	0.702	29	29	1	26	32	0.435	28	30	0.199	30	28	0.158
T3 and T4	11	9		10	10		11	9		13	7		14	6	
N stage															
N0	20	28	0.033*	23	25	0.644	22	26	0.722	24	24	0.569	21	27	0.005*
N1-N3	20	10		16	14		15	15		17	13		23	7	
Clinical stage															
I and II	16	20	0.266	18	18	1	15	21	0.348	16	20	0.186	15	21	0.016*
III and IV	24	18		21	21		22	20		25	17		29	13	

*P values showing statistically significance were indicated by*

### Assessment of survival by COX regression analysis

#### Univariate COX regression analyses and Kaplan-Meier survival curves

To determine the predictive value of immune cells infiltration, we performed the COX regression analysis. The results of the univariate COX regression analyses were summarized in Table 4.

**Table 4:** Univariate COX regression analysis of overall survival

Factors	SE	*P*	Exp (β)	95.0% CI	
Gender	0.328	0.677	1.147	0.603	2.181
Age	0.293	0.555	0.841	0.473	1.494
Smoking	0.297	0.451	0.799	0.446	1.431
Drinking	0.299	0.277	1.384	0.771	2.485
T stage	0.319	0.301	1.391	0.744	2.600
N stage	0.313	<0.001*	3.784	2.048	6.989
Differentiation	0.315	0.307	1.379	0.744	2.557
Clinical stage	0.304	0.007*	2.264	1.248	4.109
CD8	0.329	<0.001*	3.808	1.998	7.256
CD4	0.299	0.207	0.686	0.382	1.232
T-bet	0.294	0.639	0.871	0.489	1.551
CD68	0.296	0.293	0.733	0.411	1.308
CD57	0.383	<0.001*	7.718	3.646	16.338

*P values showing statistically significance were indicated by*

Univariate analyses in all patients confirmed that both late clinical stage (*p* = 0.007) and lymph node metastasis (*p* < 0.001) were associated with a shorter survival. High CD8 and CD57 expression were significantly associated with longer survival (*p* < 0.001) in our cohort.

**Fig. 2:**
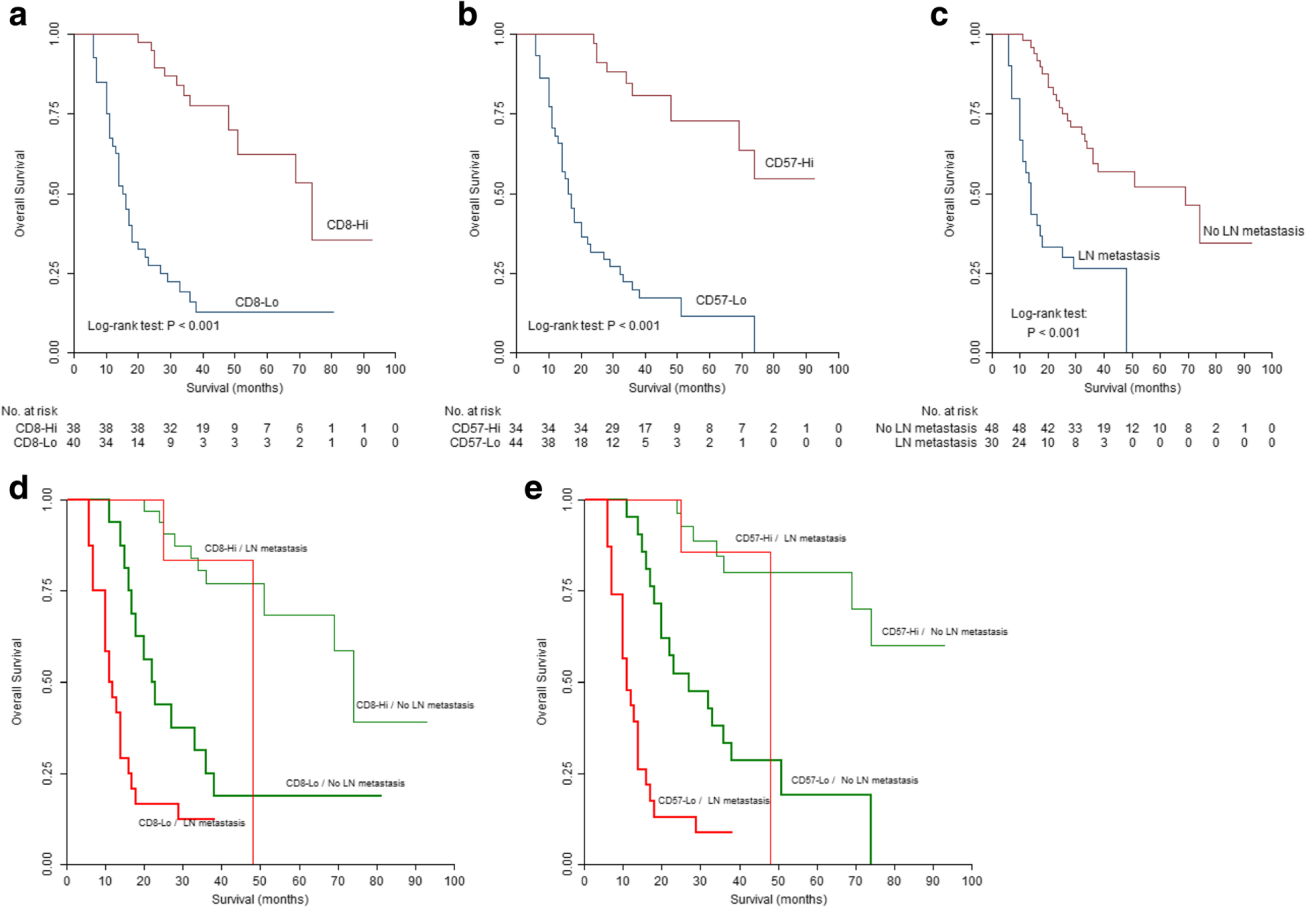
Correlation between immune cells infiltration and OS of OSCC patients. **a**, **b** and **c** Survival curves were stratified by CD8, CD57 and N stage with the Kaplan-Meier method. High CD8, high CD57 and no lymphnode metastasis were associated with longer survival (*p* < 0.001). **d**, **e** Kaplan-Meier curves illustrate the duration of survival according to the N stages and to the density of CD8^+^ cells (**d**) and CD57^+^ cells (**e**). CD8 and CD57 expression was associated with survival regardless of N stages (CD8-Lo/No LN metastasis vs. CD8-Hi/No LN metastasis, *p* < 0.001; CD8-Lo/ LN metastasis vs. CD8-Hi/ LN metastasis, *p* = 0.002; CD57-Lo/No LN metastasis vs. CD57-Hi/No LN metastasis, *p* < 0.001; CD57-Lo/ LN metastasis vs. CD57-Hi/ LN metastasis, *p* < 0.001). Hi, high expression; Lo, low expression; LN, lymphnode

Figure 2a–c shows the Kaplan-Meier survival curves based on N stages and infiltration level (high and low defined by the average number) of CD8^+^ and CD57^+^ cells. Patients with lymphnode metastasis showed shorter survival compared with those without lymphnode involvement. The average OS of patients with different levels of immune cells were: 21.38 months in low and 43.68 months in high CD8 expression group; 22.14 months in low and 46.2 months in high CD57 expression group. Comparison of the Kaplan-Meier curves for OS indicated that higher CD8 and CD57 expression were associated with better patient survival. CD4, T-bet and CD68 levels did not show any correlation with patient outcome.

We determined whether CD8 and CD57 could discriminate patient outcome at different N stages. Patients were stratified according to N stage (N0 and N1–3). As Fig. 2d and e showed, a strong stroma CD8 and CD57 expression correlated with favorable prognosis regardless of invasion of regional lymph nodes (*p* < 0.01).

#### Multivariate COX regression analysis.

The results of multivariate COX regression analysis confirmed that lymph node involvement (*p* = 0.008), CD8 expression (*p* = 0.03) and CD57 expression (*p* < 0.001) were significantly correlated with OS (Table 5). CD8 and CD57 expression can be considered as independent prognostic factors in OSCC patients. Clinical stage was not statistically significant in multivariate analysis.

**Table 5:** Multivariate COX regression analyses of 78 OSCC patients

Factors		SE	*P*	Exp (β)	95% CI	
T stage	T1/T2			Reference		
	T3/T4	0.487	0.529	1.359	0.523	3.530
N stage	N0			Reference		
	N1-N3	0.602	0.008*	4.969	1.527	16.168
Clinical stage	I and II			Reference		
	III and IV	0.667	0.282	0.488	0.132	1.804
CD8	Low	0.358	0.030*	2.174	1.078	4.384
	High			Reference		
CD4	Low	0.328	0.909	1.038	0.545	1.977
	High			Reference		
T-bet	Low	0.310	0.836	1.066	0.580	1.959
	High			Reference		
CD68	Low	0.316	0.177	1.533	0.825	2.848
	High			Reference		
CD57	Low	0.441	<0.001*	6.576	2.768	15.623
	High			Reference		

*indicated P values with statistically significance

### Predictive accuracy for OS

**Fig. 3:**
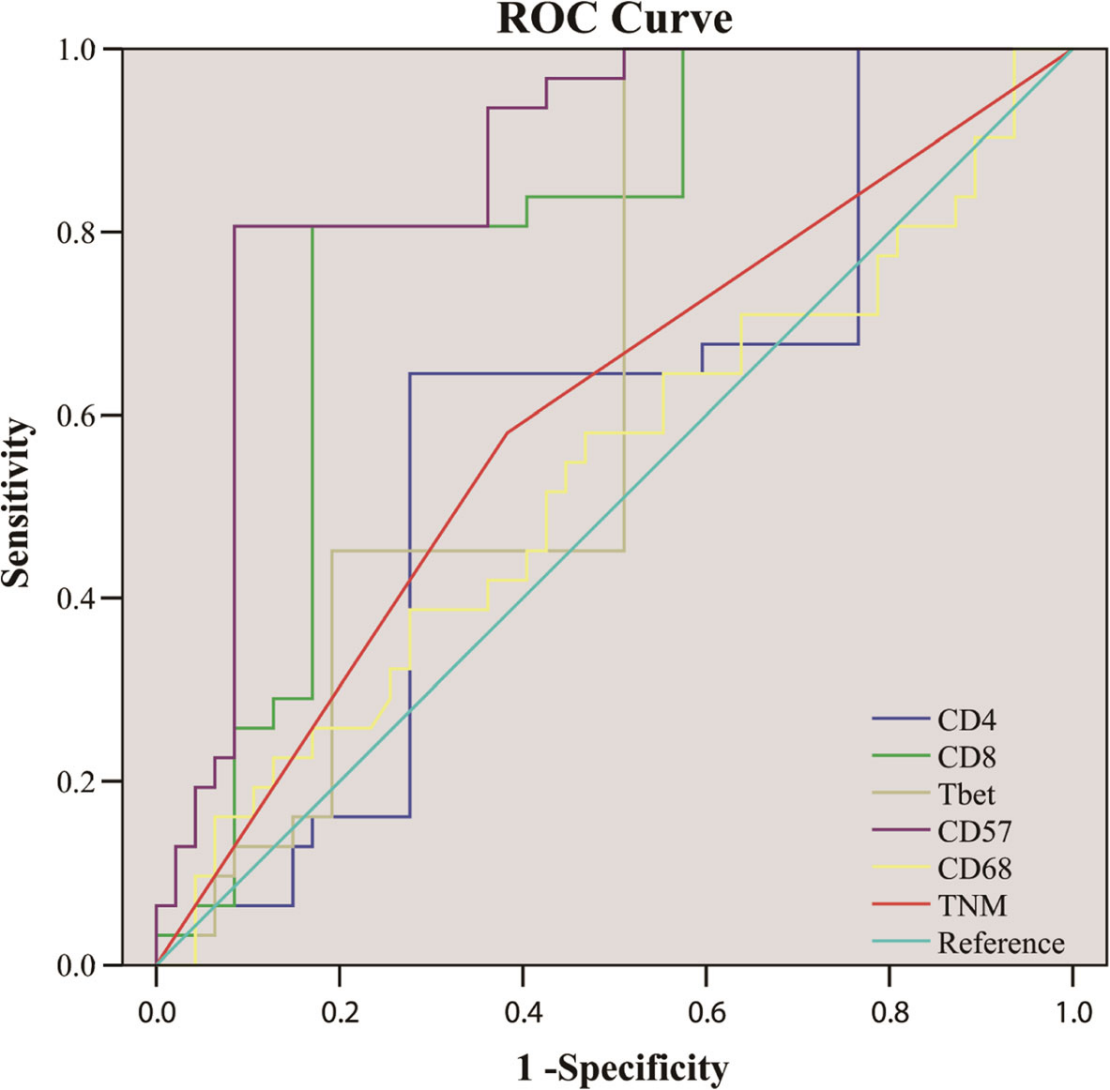
ROC curves indicating predictive accuracy, sensitivity and specificity of each potential parameter. AUCs of CD8 and CD57 were 0.784 (95% CI 0.680–0.889) and 0.868 (95% CI 0.785–0.950) respectively, significantly higher than TNM staging (AUC 0.599, 95% CI 0.469–0.728)

To determine the predictive accuracy of immune cells on OS, we performed ROC curve analyses. As shown in Fig. 3 and Table 6, the respective predictive accuracy of CD8 and CD57 expression were all superior to TNM staging in determining patient outcome. CD57 provided the highest predictive accuracy (AUC = 0.868; 95% CI, 0.785–0.950).

**Table 6:** Summary of the OS predictive accuracy of immune cells

Predictive factors	AUC	SE	*P*	95% CI	
TNM	0.599	0.066	0.141	0.469	0.728
CD8	0.784	0.053	0.000	0.680	0.889
CD4	0.581	0.067	0.226	0.450	0.713
T-bet	0.651	0.063	0.024	0.528	0.774
CD68	0.533	0.068	0.624	0.399	0.667
CD57	0.868	0.042	0.000	0.785	0.950

*SE* standard error, *95% CI* 95% confidence interval

## Discussion

A comprehensive detection and assessment of factors influencing prognosis is important for improving patient management of OSCC. This study performed on various immune parameters in OSCC patients has shown that infiltration levels of immune cells were important predictive factors for patient outcome. Particularly, among these, stroma CD8 and CD57 expression were found to be the most powerful prognostic indicators.

We found that patients with strong CD8 expression had a significantly better clinical outcome. CD8^+^ cell is a crucial component of cell-mediated immunity as it produces interferon-γ upon interaction with tumour targets. In agreement to our findings, strong tumour infiltration by CD8^+^ cells has been correlated with a favorable outcome in several tumor types [31–36], including head and neck cancer [14–17, 37]. Former study on colorectal cancer with a large cohort has shown that infiltrating CD8^+^ T cells had a prognostic value that superior to and independent of those of TNM classification [38]. In our study, CD8 expression was significantly higher in patients with no lymph node involvements, when stratified patients according to N stages, CD8 expression was still correlated with favorable prognosis regardless of N stages, which revealed the superior prognostic value of CD8 expression.

Detailed analysis of CD57^+^ inflammatory cell activity in the host immune systems and head and neck cancer development has not been well defined. Function of tumor infiltrated CD57^+^cells remain unclear. Former studies have shown that CD57 was not an independent factor associated with survival [39, 40]. A more recent study included 57 cases of OSCC indicated that high level of CD57^+^ cells correlated with longer OS in OSCC patients and CD57 expression could be considered as a powerful indicator of OS [41]. This result was accordant with our study. In the present study, high CD57 expression was significantly associated with early clinical stage and no lymph node metastasis. A strong CD57 expression was found to be an independent prognostic factor for longer survival, moreover, it represented better predictive value compared with other immune cells, including CD4 and CD8 positive cells, and TNM staging. As researchers have reported positive correlation between presence of CD57^+^ T cells and cancer progression [25], our results indicated the potential important role of CD57^+^ NK cells in anti-tumor immunity against OSCC, which remained to be further confirmed by co-staining for CD3.

CD4^+^ cells serve a variety of biological functions according to their subpopulations. Th1 assist cytotoxic function of CD8^+^ cells, while Th2, Th17 and regulatory T cells (Tregs) could negatively regulate the adaptive immunity. T-bet plays critical roles in the differentiation of Th1 and regulates the Th1/Th2 shift. In OSCC, the role of CD4 remains controversial as mixed findings have been reported. Balermpas P et al. found no correlation between CD4 expression and clinical outcome of patients with head and neck cancer [15], whereas Nguyen N et al. reported that higher CD4 levels predicted improved OS and disease-specific survival [17]. T-bet has been shown to be associated with better outcome in patients with renal cell cancer, breast cancer, gastric cancer and colorectal cancer [19, 42–45]. We did not observe any correlation for either CD4 or T-bet expression and clinical outcome in our series. As CD4^+^ cells consist of various subpopulations, each of them could affect tumor behavior. The clinical significance according to each phenotype remains to be established.

Another important component of innate immunity, macrophages, is functionally differentiated into pro-inflammatory “M1” and alternative anti-inflammatory “M2” phenotypes. M1 type, commonly identified by staining the CD11c antigen, was conferred a significantly better prognosis. M2 type, expressing CD163 and MRC1, promoted tumor growth, invasion, angiogenesis, and metastasis [46]. Generally speaking, CD68 is the best established marker of tumor associated macrophages (TAMs), it is expressed on both M1 and M2 phenotypes. Ni YH et al. found that CD68^+^ TAMs infiltration in tumor stroma was correlated with high tumor grade and lymph node metastasis. More TAMs was correlated with short OS, but TAMs was not an independent predictive factor [46]. Our study showed that CD68 expression was not significantly associated with OSCC patient survival in both univariate and multivariate analysis, which was consistent with a more recent study including larger cohort of 278 patients by Nguyen N et al. [17] This result indicated the counterweight of M1 and M2 in tumor microenvironment of OSCC. Further study is required to differentiate M1 and M2 to determine their respective functions in OSCC.

In this study, special attention was paid for evaluating immune infiltrates. Regarding localization of the stained markers, stroma and tumor periphery not tumor nest were evaluated, because most current studies have shown that stroma immune cells were superior and more reproducible [47, 48]. We used full section over core biopsy (such as in the settings of tissue microarrays) because up till now, there was no published evidence that Tissue Microarrays (TMAs) can mirror or reflect the potential heterogeneity of immune cells in tumor, and the number and diameter of cores in TMAs vary, which will likely affect the accuracy needed for the determination of various immune components [30]. Regarding the scoring system, a quantitative parameter of cells count per HPF was applied. Machine scoring approaches, while promising, have not been published or validated yet, which needs to be explored in future studies.

There were some limitations in the current study. First was the limited size of our cohort with all patients being Chinese OSCC patients. It remains unclear if Chinese ethnic background is associated with particular immunological features. Second, a critical negatively regulated factor of immune response, Tregs, has not been separately stained. As a subpopulation of CD4^+^ cells, Tregs might contribute to the final negative results of association between CD4 and survival. Third, as CD68 expression cannot differentiate M1 and M2 subtypes of TAMs, further study should be designed including reliable markers of M1 and M2 TAMs for precise analysis. Fourth, without co-staining for CD3, the specific functions of CD57^+^ T cell and CD57^+^ NK cell in OSCC were not illuminated.

## Conclusions

In conclusion, our study revealed that stroma CD57 and CD8 expression were independent prognostic markers for OS of OSCC patients. When compared with TNM staging, expression of CD8 and CD57 provided superior predictive function. The results from this small OSCC cohort suggest that tumor infiltrating immune cells can potentially predict patient survival, thus provide new clues to therapeutic strategies in OSCC based on utilizing host immune response.

## Abbreviations

AUC: Area under the curve
CTLs: Cytotoxic lymphocytes
HPF: High-power field
NK cells: Natural killer cells
OS: Overall survival
OSCC: Oral squamous cell carcinoma
ROC: Receiver operating characteristic
TAMs: Tumor associated macrophages
TMAs: Tissue microarrays
Tregs: Regulatory T cells
